# Voltage-Gated Sodium Channels Regulate the Migration Potential of Human Endometrial Mesenchymal Stem/Stromal Cells in 2D and 3D Culture

**DOI:** 10.3390/cells15100851

**Published:** 2026-05-07

**Authors:** Margarita Shamatova, Mariia Shorokhova, Irina Vassilieva, Vladislav Chubinskiy-Nadezhdin, Anastasia Sudarikova

**Affiliations:** 1 Institute of Cytology, Russian Academy of Sciences, 194064 St. Petersburg, Russiashili-mariya@yandex.ru (M.S.); irinaovas@yandex.ru (I.V.);; 2 Shemyakin-Ovchinnikov Institute of Bioorganic Chemistry, Russian Academy of Sciences, 117997 Moscow, Russia

**Keywords:** voltage-gated sodium channels, endometrial mesenchymal stem/stromal cells, cell migration, veratridine, 3D cell culture, cell spheroids

## Abstract

Human endometrial mesenchymal stem/stromal cells (eMSCs) are widely used in laboratories and clinical applications to study various aspects of tissue engineering and regenerative medicine. Three-dimensional (3D) cultivated MSCs have a higher therapeutic efficacy compared to 2D culture. Ion channels are involved in maintaining many physiological cell functions, including proliferation, differentiation, apoptosis, and migration. This study describes the functional expression of voltage-gated sodium channels (NaV) in eMSCs and the role of these channels in cell migration. Using RT-PCR analysis and immunofluorescent microscopy, we identified the expression of almost all pore-forming alpha (NaV 1.1, 1.2, 1.4–1.9) and channel-modulating beta-NaV subunits (except beta2) in eMSCs. In the whole-cell patch-clamp configuration, channels activated by membrane depolarization of eMSC were detected. The channels were blocked by the selective NaV antagonist TTX in nanomolar concentrations. The NaV agonist veratridine at a concentration of less than 40 μM inhibited voltage-gated sodium currents, while 100 μM and above prevented channel inactivation. The wound healing assay showed that both TTX (10 μM) and veratridine (100 μM) reduced the migration properties (the wound healing rate) of eMSCs cultivated in 2D conditions compared to the control. An opposite effect by both agents was shown on the motility of eMSCs cultivated in 3D conditions, increasing the cell spreading rate from spheroids. Our data suggest that NaV channels are expressed in human eMSCs and play an important role in the regulation of stem cell migration; this regulatory mechanism significantly depends on the culture conditions of MSCs.

## 1. Introduction

Human endometrial mesenchymal stem/stromal cells (eMSCs) are a unique population of postnatal stem cells localized in the basal and functional layers of the uterine mucosa [1]. eMSCs possess the classic characteristics of mesenchymal stem cells: high proliferative and expansive potential, the ability to undergo multilineage differentiation (in the adipogenic, osteogenic, and chondrogenic directions), and pronounced immunomodulatory and trophic properties [1,2]. Due to the availability of source material (menstrual blood) and the absence of significant ethical restrictions, eMSCs are considered a promising autologous cellular resource for tissue bioengineering and regenerative medicine [2]. eMSCs are also widely used for the fundamental study of physiological and pathophysiological processes in stem cells.

Traditionally, most laboratory studies aimed at assessing the functional processes of eMSCs are conducted under two-dimensional (2D) culture conditions, where cells form a monolayer on a plastic substrate. Despite the technical simplicity and reproducibility of the method, increasing evidence suggests that the 2D model does not reflect actual cellular behavior in vivo. Under flat adhesion conditions, artificial cell flattening, polarization of the apical-basal region of receptors, and changes in cytoskeletal rigidity occur; as a result, distortion of intracellular signaling pathways regulating proliferation, differentiation, and motility also occurs [3]. The transition to three-dimensional (3D) culture systems, such as spheroids, allows for the recreation of the natural microenvironment between cells. Cells in a 3D environment exhibit a morphology characteristic of fibroblast-like cells in situ, form complex intercellular contacts, and interact with extracellular matrix components in all directions, which fundamentally alters gene expression and proteomic profiles [3,4]. It has been shown that MSCs from spheroids survive better after transplantation and are more effective in stem cell-based therapies due to the enhanced secretion of different growth factors, signaling molecules, etc., involved in the regenerative processes [5,6].

The study of ion channels, which are transmembrane proteins, in eMSCs is a relevant task since they are involved in the maintenance of many physiological functions of the cells, including proliferation, differentiation, apoptosis and migration. Voltage-gated Na^+^ channels (NaV) are widely expressed in excitable cells (nerve and muscle) and play an important role in the generation and propagation of action potentials, the transmission of nerve signals, and the regulation of muscle contraction [7]. NaV consists of one α subunit that is responsible for the functioning of the channels in the membrane, and one or two β subunits that modulate channel activity or can perform some non-conducting function, such as serving cell-adhesion or cytoskeleton-binding molecules etc. [8,9]. NaV subunits have been shown to be expressed in various non-excitable cells, including stem cells, macrophages, lymphocytes, astrocytes and different cancer cells and can regulate cell growth, invasion (motility), and metastasis [10,11,12]. However, their role in non-excitable cells is not fully understood.

Ion channel gene expression profiles may vary among different stem cells depending on the species, origin, sources, or cultivation conditions [13,14]. Recently, it has been considered that three-dimensional (3D) cultivated MSCs as cellular spheroids are closer to physiological conditions in vivo compared to those in 2D culture. In previous work from our laboratory, several types of functional ion channels were identified in eMSCs, including sodium-selective ENaC channels [15], calcium-activated potassium BK channels [16], and mechano-gated calcium-permeable Piezo1 channels [17]. Moreover, the role of Piezo1 in the migration of eMSCs cultured under canonical 2D conditions or 3D spheroids [14,18] was established. Furthermore, Piezo1 was found to regulate the formation and spreading of eMSCs from spheroids. A question arises about the possible involvement of NaV in the functioning of eMSCs cultivated in different conditions.

In the present study, the expression pattern of NaV subunits, their functional activity, inhibition by the selective antagonist tetrodotoxin (TTX), and modulation by the agonist veratridine were studied in human 2D eMSCs. We also investigated the potential role of NaV in the migration properties of eMSCs in 2D and 3D culture and provided evidence of the involvement of NaV in the regulation of eMSCs’ motility. Our data indicate that before using various agents in stem cell-based therapy, the possibility of their different or opposite effects on cells in 2D and 3D culture should be considered.

## 2. Materials and Methods

### 2.1. Cells

The human endometrial mesenchymal stem/stromal cell line (eMSCs) was provided from the stem cell collection of the Department of Intracellular Signaling and Transport (Institute of Cytology, St. Petersburg, Russia). These cells were isolated from desquamated endometrium collected from the menstrual blood of healthy women and were previously characterized as follows: express the surface markers CD105, CD44, CD73, CD90, CD146, and CD29; are negative for the hematopoietic markers CD34 and CD45; and have the ability to differentiate into adipocytes, chondrocytes, and osteoblasts [19]. eMSCs met the International Society for Cell Therapy criteria for multipotent MSCs [20]. The expression of CD markers was also confirmed in our previous work [16].

eMSCs were cultured in DMEM/F12 growth medium (Gibco, Waltham, MA, USA) supplemented with 10% fetal bovine serum (FBS, HyClone, Pasching, Austria), 1% Glutamax (Gibco, Waltham, MA, USA), and 1% penicillin–streptomycin (Gibco, Grand Island, NY, USA) at 37 °C in a humidified chamber with 5% CO_2_. The cell line was free of mycoplasma contamination for the described experiments. Cells were passaged weekly at a 1:3 ratio with 0.05% EDTA–trypsin solution (Gibco) for 6–14 passages. One to two days before the experiment, 2-dimensional (2D) cells were seeded onto 4 × 4 mm or 10 × 10 mm coverslips depending on the type of experiment.

### 2.2. Formation of Spheroids (3D Culture)

For the formation of spheroids (3D culture), a suspension of eMSCs (2D culture) was cultured using the hanging drop method as previously described [18]. Briefly, cells were dissociated using a 0.05% trypsin-EDTA solution (Gibco), and the enzymatic reaction was neutralized by adding complete growth medium DMEM/F12 (supplemented with 1% Glutamax and 1% penicillin–streptomycin, all from Gibco, Waltham, MA, USA). Cells were counted using a Countess II automated cell counter (Thermo Fisher Scientific, Waltham, MA, USA). A total of 35 µL drops of complete DMEM/F12 medium containing 2 × 10^5^ cells/mL (7000 cells) were placed on the lid of 10 cm diameter Petri dishes (Jet Biofil, Guangzhou, China), then the lid was inverted and incubated for 48–72 h at 37 °C and 5% CO_2_; the cells aggregated into multicellular spheroids under the gravity-enforced self-assembly.

### 2.3. RT-PCR

Total RNA was isolated from eMSCs using the RNeasy mini kit (Qiagen, Hilden, Germany). A commercially available kit with MMLV reverse transcriptase (Sileks, Moscow, Russia) was used to perform reverse transcription. The Taq PCR Core Kit (Qiagen) was used for PCR. The RT-PCR sample (per 15 μL mixture) consisted of 0.3 μL dNTPs, 1.5 μL 10× Hot-taq CoralLoad buffer (with dye), 0.08 μL Hot-taq polymerase, 0.75 μL each of forward and reverse primers, 1.5 μL cDNA, and 10.12 μL water. The list of primers specific for NaV subunits (Synthol, Moscow, Russia) is presented in Table 1. For amplification on a T100 Thermal Cycler (Bio-Rad, Hercules, CA, USA), the following protocol was used: 94 °C for 9 min 30 s, then 34 cycles (94 °C for 40 s, 55 °C for 30 s, 72 °C for 30 s) and 72 °C for 5 min. Electrophoresis was performed in a 6% polyacrylamide gel in a Bio-Rad chamber (Bio-Rad, Hercules, CA, USA). The gel was stained with GelRed dye (Biotium, Fremont, CA, USA). The results were visualized using the E-Gel Imager system (Thermo Fisher Scientific, Waltham, MA, USA).

### 2.4. Immunofluorescence Staining

To detect the expression of NaV proteins in eMSCs, immunofluorescence staining with specific primary anti-Pan NaV antibodies (ASC-003, Alomone Labs, Jerusalem, Israel, RRID:AB_2040204) against the NaV 1.1 intracellular epitope that is the same in all NaV1 subunits was carried out according to the standard procedure [21]. Anti-SCN1B antibodies against an extracellular epitope of NaV β1 (ASC-041, Alomone Labs, RRID:AB_2341071) were used to detect the β1 subunit in eMSCs. For 2D culture, cells were seeded on coverslips 1–2 days before the beginning of the experiments; for 3D culture, spheroids were seeded on poly-DL-lysine-precoated coverslips 2 h before the staining. Briefly, eMSCs in both 2D and 3D cultures were fixed with 4% paraformaldehyde for 15 min, permeabilized with 0.25% Tween-20 for 10 min (for intracellular antibodies), and incubated with 10% goat serum for 1 h to protect from nonspecific binding. Then, cells were incubated with primary antibodies (1:100) at +4 °C overnight and then with secondary antibodies (1:400, 1 h, goat anti-rabbit Cy3, Thermo Fisher Scientific, Waltham, MA, USA). The cell membrane marker (ganglioside GM1) was labeled with a FITC-conjugated cholera beta subunit (5 µg/mL FITC-CTb, 15 min at 4 °C). Cell nuclei were stained with DAPI (Sigma-Aldrich, Burlington, MA, USA; up to 30 min in the dark). For control, eMSCs were incubated without primary antibodies. The studied proteins were visualized on a confocal microscope Olympus FV3000 (Olympus, Shinjuku, Tokyo, Japan, RRID:SCR_017015) using a 40×/1.3 NA objective and analyzed using ImageJ 1.47v software (National Institutes of Health, Bethesda, MD, USA).

### 2.5. Electrophysiology

The activity of voltage-gated ion channels in eMSCs was recorded using the patch-clamp technique in the whole-cell configuration. The pipettes were made of a thin-wall BF-150-110-10 borosilicate glass (Sutter Instruments, Novato, CA, USA) on a P-97 puller (Sutter Instrument, Novato, CA, USA). The resistance of the pipettes filled with solution was 4–8 MΩ. Ion currents were recorded using an Axopatch 200B amplifier (Molecular Devices Corp., San Jose, CA, USA, RRID:SCR_018866), an Axon Digidata 1550B analog-to-digital converter (Molecular Devices Corp., San Jose, CA, USA), a motorized micromanipulator (PatchStar, Scientifica, Uckfield, East Sussex, UK), an inverted microscope, and a personal computer. The following protocol was used to activate and record ion currents: a voltage of −100 mV was maintained across the membrane for 4 s, then the membrane potential was changed stepwise from −100 mV to +50 mV (with an increment of 10 mV) at 40 ms intervals. Tetrodotoxin (TTX) was diluted to the intermediate stock solution of 1 mM in water, veratridine (Sigma-Aldrich, Burlington, MA, USA) was stocked as 50 mM in dimethyl sulfoxide (DMSO) and then diluted to the needed concentration with the extracellular solution before use. Currents were recorded and analyzed using pClamp 10.7 software (Molecular Devices Corp., San Jose, CA, USA, RRID:SCR_011323), Microcal Origin 9.1 (Microcal Software, Northampton, MA, USA, RRID:SCR_002815), GraphPad Prism 8.0 (GraphPad Software, San Diego, CA, USA, RRID:SCR_002798) and Microsoft Excel (MS Corporation, Seattle, WA, USA, RRID:SCR_016137).

In whole-cell patch-clamp experiments, we used an extracellular (bath) solution containing: 145 mM NaCl, 1 mM MgCl_2_, 2 mM CaCl_2_, 10 mM HEPES/TrisOH, pH 7.3; an intracellular (pipette) solution was: 135 mM CsAspartate, 5 mM NaCl, 10 mM glucose, 10 mM EGTA, 1 mM MgCl_2_, 10 mM HEPES/TrisOH, and 0.176 mM CaCl_2_ to maintain the free 0.01 µM Ca^2+^ concentration (pCa8).

### 2.6. Wound Healing Assay

Cell migration properties were studied using the experimental wound healing assay. To form wounds, eMSCs were seeded into special Ibidi silicone inserts (Ibidi, Gräfelfing, Germany, 30,000–40,000 cells per side) placed in 4- or 24-well plates. The plates were then placed in a CO_2_ incubator (37 °C, 5% CO_2_) overnight to form a cell monolayer. Immediately before the experiment, the silicone inserts were removed, imitating the wound, and the culture medium in each well was replaced with fresh DMEM/F12 with 10% FBS or a medium containing a reagent (veratridine or TTX). Phase-contrast images of wounds at the beginning of the experiment (0 h), 24 h, and 42 h were obtained using an inverted microscope (10× objective, Micromed, St. Petersburg, Russia, or AxioObserver Z1, Carl Zeiss, Oberkochen, Germany) and a Toupcam 5.1 digital camera (Touptek Photonics, Hangzhou, China) controlled by Toupview 3.7 software (Touptek Photonics, Hangzhou, China). Wound areas were calculated manually using the Area Measurement tool in ImageJ (NIH, Bethesda, MD, USA) and normalized to the initial wound size (0 h). All experiments were carried out in triplicate.

### 2.7. Metabolic Activity Test

The effect of TTX or veratridine on the viability and proliferation of eMSCs was assessed using the standard colorimetric 3-(4,5-dimethylthiazol-2-yl)-2,5-diphenyltetrazolium bromide (MTT) test at 24 and 48 h time intervals according to the manufacturer’s protocol (Servicebio, Wuhan, China). Five thousand cells (per 0.1 mL of full medium) were placed in each well of 96-well plates (NEST, Wuxi, China) and allowed to attach to the surface (for 3 h) before the addition of the reagents. A total of 20 μL of MTT was added after 20 h and 44 h and incubated for 4 h. For each time point, 6 experimental replicates (wells) were used with each of the reagents. DMSO (0.2%) was used as a negative control. After incubation, the medium was removed, the pellet was dissolved in 100 μL of DMSO, incubated for 10 min at 37 °C and thoroughly mixed. The optical density (OD600) was measured using a microplate reader (BK-EL10C, Biobase, Jinan, China) at an absorbance of 600 nm. Background value (OD600 in 100 μL of DMSO) was subtracted from each value.

### 2.8. Spheroid Spreading Assay

Spheroid spreading is the process of cell dispersal (“escape”) from spheroids deposited on a flat adhesive surface. To determine the role of NaV channel activity in eMSC spheroid spreading, each droplet containing a formed spheroid was manually transferred to a separate well of a 24-well plate, and the spheroids were allowed to adhere to the surface for 2 h. Since only one spheroid was present per well, we analyzed each spheroid individually. Reagents affecting NaV channels (TTX, 10 μM, veratridine, 100 μM) were then added to the wells; for the veratridine control, 0.2% DMSO was added to the medium. Images of the spheroids were acquired using an inverted microscope AxioObserver Z1 (Carl Zeiss, Oberkochen, Germany), equipped with a chamber in which 37 °C and 5% CO_2_ was maintained, and a CCD camera after spheroid attachment (2 h) and every 2 h for 24 h from the start of the experiment. Using these images, the areas of the spheroids, including the cells that migrated from the spheroid mass, were determined using the Measure tool in FIJI 1.54p software. The spheroid spreading rate was then determined as the ratio S(N)/S(0), where S0 is the initial area of the original spheroid (2 h after transfer to a flat surface), and S(N) is the area of the migrated cells from the spheroid for every 2 h up to 24 h. All experiments were carried out in triplicate.

### 2.9. Membrane Potential Assay

To compare the resting membrane potential, the 2D and 3D cell cultures were plated on glass coverslips 24 h before the experiments. Then, the cells were loaded in serum-free DMEM/F12 for 30 min at 37 °C with 1 µM of potential-sensitive bis-(1,3-dibutylbarbituric acid)trimethine oxonol (DiBAC4(3)) dye that enters the cells in response to membrane depolarization, thus increasing the fluorescence (Thermo Fisher Scientific, Eugene, OR, USA). Due to the obvious problem of correct fluorescent measurements from the tightly packed multicellular aggregates, the fluorescent signals were recorded from the cells that migrated from the spheroid mass.

The coverslips with the cells were placed on the experimental camera filled with a quasi-physiological Na^+^-based solution containing (in mM) 145 NaCl, 2 CaCl_2_, 1 MgCl_2_, 10 HEPES/TrisOH, pH = 7.3. The camera was mounted on the stage of AxioObserver Z1 inverted microscope (Carl Zeiss, Oberkochen, Germany) equipped with a Zeiss Colibri diode light source, a Plan-Apochromat 20×/0.8 NA objective and a Zeiss AxioCam HR3 CCD camera controlled with Zeiss Axiovision 4.8.2 software. The diode excitation wavelength was 488 nm; the emission was collected at 500–550 nm. The working concentration of DiBAC4(3) was prepared from its DMSO stock (2 mM) at 1:2000 dilution; the amount of vehicle was 0.05%. According to the protocol, 1 µM of diBAC4(3) was present in all solutions. The basal DiBAC4(3) fluorescence in Na^+^-based bath solution was captured for 1–2 min at 1 frame per 5 s, and then the solution was changed to K^+^-based containing (in mM): 145 KCl, 2 CaCl_2_, 1 MgCl_2_, 10 HEPES/TrisOH, pH = 7.3 to depolarize the cells by nullifying their membrane potential. The camera exposure time was kept constant during the recordings. As DiBAC4(3) is a slow-response probe, the fluorescent signals in response to depolarization were captured until their stabilization (reaching the plateau), indicating the stoppage of DiBAC4(3) entry to the cytoplasm. Three independent experiments on 2D and 3D cultures were conducted, and at least 50 cells in each experiment were used in the analysis. The time-lapse image stacks were analyzed in ImageJ (NIH, Bethesda, MD, USA). Cells were outlined using the freehand selection tool, and each cell was added as a region of interest (ROI) to the ROI manager (“Analyze—ROI manager—add ROI”). Then, a mean gray value in each ROI was measured using the “Multi-Measure” command. The region containing no cells (background fluorescence) was selected, and values in this region were subtracted from the signals. The fluorescence values were normalized to the first frame (F_0_), and the data are presented as F/F_0_ (± S.D.), where F is the mean fluorescence at each time point.

The relative degree of resting membrane potential change in 2D and 3D culture was assessed by calculation of the FKCl/FNaCl value, where FNaCl is the mean DiBAC4(3) fluorescence in an Na^+^-based solution (before application of K^+^-based solution) and FKCl is the mean DiBAC4(3) fluorescence at the last time point in the experiment (DiBAC4(3) equilibration). The FKCl/FNaCl reflects the degree of the difference between resting membrane potential and 0 mV: high FKCl/FNaCl indicates a high degree of cell hyperpolarization.

### 2.10. Statistics

For statistical analysis, GraphPad Prism 8.0 (GraphPad Software, San Diego, CA, USA, RRID:SCR_002798) and Microsoft Excel (MS Corporation, Seattle, WA, USA, RRID:SCR_016137) were used. The absence of outliers in each data set was confirmed by Grubbs’ test (alpha = 0.05) or the ROUT method (Q = 1%). Before comparisons, the data were checked for normality (Shapiro–Wilk test, *p* = 0.05). Data were compared using a paired or unpaired Student’s *t*-test or Wilcoxon test, depending on the experiment; *p* < 0.05 was considered significant. n is the number of experiments.

## 3. Results

### 3.1. NaV Expression Pattern in eMSCs

**Figure 1:**
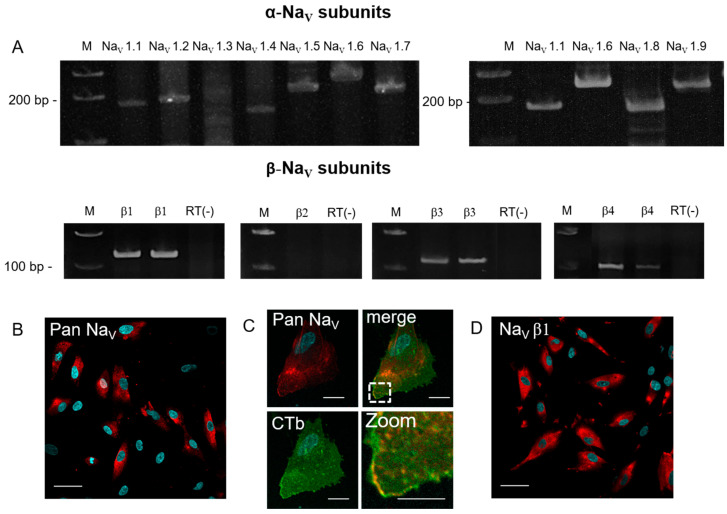
Expression of various NaV channel subunits in eMSCs. (**A**) mRNA of NaV α-subunits was detected by RT-PCR: SCN1A (NaV 1.1, 178 bp), SCN2A (NaV 1.2, 192 bp), SCN4A (NaV 1.4, 148 bp), SCN5A (NaV 1.5, 208 bp), SCN8A (NaV 1.6, 247bp), SCN9A (NaV 1.7, 196 bp), SCN10A (NaV 1.8, 168 bp), SCN11A (NaV 1.9, 233 bp) and beta subunits: SCN1B (NaV β1, 125 bp), SCN2B (NaV β2, 134 bp), SCN3B (NaV β3, 136 bp), SCN4B (NaV β4, 96 bp). M—DNA size marker. RT(-)—negative control without reverse transcriptase. (**B**) Immunofluorescence staining of NaV (Pan) and (**D**) β1-subunit proteins is presented in the red channel; cell nuclei were stained with DAPI (blue), scale bar: 50 μm. (**C**) Representative confocal images (scale bar is 20 μm) of the cell stained with DAPI (blue), NaV (Pan, red), CTb (green, cell membrane marker ganglioside GM1) and the corresponding higher magnification image (Zoom, scale bar is 10 μm) indicate the presence of NaV (Pan) in the plasma membrane.

In order to determine the presence of NaV in eMSCs, firstly, RT-PCR experiments were performed. The expression pattern of almost all NaV isoforms was investigated, including nine pore-forming α subunits and four β subunits. RT-PCR analysis revealed the presence of mRNA of the following NaV isoforms: α-subunits for NaV 1.1, 1.2, 1.4–1.9, β-subunits for NaV 1, 3, 4. The mRNA expression of SCN1A (NaV 1.3) and SCN2B (NaV β2) was not detected (Figure 1A).

The expression of NaV proteins in eMSCs was confirmed using immunofluorescence microscopy experiments with specific primary antibodies. Since it is inappropriate to test the expression of each NaV subunit, we used an Anti-Pan NaV antibody against the epitope common to all NaV subunits; we also confirmed the expression of the β1 subunit using an extracellular antibody against NaV β1 (Figure 1B).

### 3.2. Functional Activity of Voltage-Activated Sodium Channels in the Membrane of eMSCs

**Figure 2:**
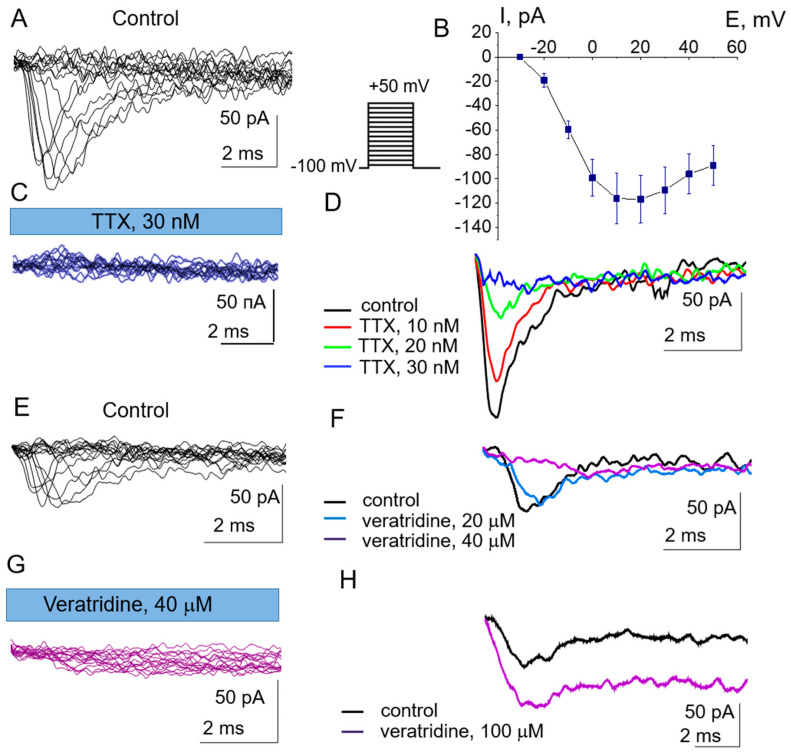
Functionally active NaV channels in the plasma membrane of eMSCs. (**A**) An example of currents activated by membrane depolarization is shown. Currents in the whole-cell configuration were recorded. The membrane potential was maintained at −100 mV for 4 s, then varied from −100 mV to +50 mV (in 10 mV steps) over 40 ms (see scheme). (**B**) Mean current-voltage relationship calculated for 9 independent experiments. (**C**) Effect of the NaV blocker TTX (30 nM) on inward voltage-dependent currents. (**D**) Effect of different TTX concentrations (10, 20, and 30 nM) on sodium currents at a potential of +10 mV. (**E**) Recordings of currents activated by membrane depolarization and (**F**,**G**) the effect of different concentrations of veratridine on the channels at +10 mV. (**H**) Effect of veratridine (100 μM) on inward voltage-dependent currents.

Possible functioning of NaV channels in the plasma membrane of eMSCs was studied using the patch-clamp method in the whole-cell configuration. Inward ion currents were detected upon membrane depolarization according to the protocol (see Section 2, scheme in Figure 2).

Figure 2A,B show an example of current recordings and the mean current-voltage relationship (n = 9). In this experiment, sodium channels were activated at a potential of −20 mV, reaching a peak at about 20 mV. Activation of inward currents, which reached a maximum within a few milliseconds and then completely inactivated, was observed at maintained membrane potentials of −10 to −20 mV, and average peak current values of 59 ± 38 pA were recorded at potentials of +10 to +20 mV (n = 14). Thus, the kinetic properties of the recorded channels are close to NaV. The percentage of the successful experiments on eMSCs in which such activity was detected was 77% (n = 27). The channels were abolished by the selective NaV inhibitor TTX (30 nM, n = 14, Figure 2C), which proves that the observed channels belong to the NaV family. It is known from the literature that different NaV subunits have different sensitivities to TTX [22]. To determine the activity of which NaV channels we recorded in patch-clamp experiments, a series of experiments with different TTX concentrations was performed to find the minimum concentration required to block NaV (Figure 2D). We found that the concentration required to inhibit half the current for NaV is 20 nM. Thus, the observed channels are TTX-sensitive.

The effect of the steroidal alkaloid veratridine, known to affect the inactivation of NaV, on the voltage-activated sodium currents in eMSCs was studied similarly to the TTX experiments in the whole-cell configuration, using concentrations ranging from 5 to 100 μM (Figure 2E–H). We found two opposite effects of veratridine on sodium currents: (1) voltage-activated currents were blocked by veratridine with a half-maximal concentration of 20 μM. The channels were completely blocked at a veratridine concentration of 40 μM (n = 3, Figure 2G). The maximum current level was observed at maintained membrane potentials of −10 to −20 mV, and the average peak current values of 43 ± 8 pA were recorded at potentials of +10 to +20 mV (n = 4). (2) In four whole-cell experiments, we observed an increase in the channel open time after administration of veratridine (100 μM), which is the classic action of the agent on NaV channels (Figure 2H). The maximum current was observed at membrane potentials of −10 to −20 mV, and average peak current values of 72 ± 37 pA were recorded at potentials of +10 to +20 mV (n = 4).

### 3.3. Contribution of NaV to the Migration Properties of eMSCs

#### 3.3.1. TTX and Veratridine Reduce the Migration Potential of eMSCs in 2D Culture

**Figure 3:**
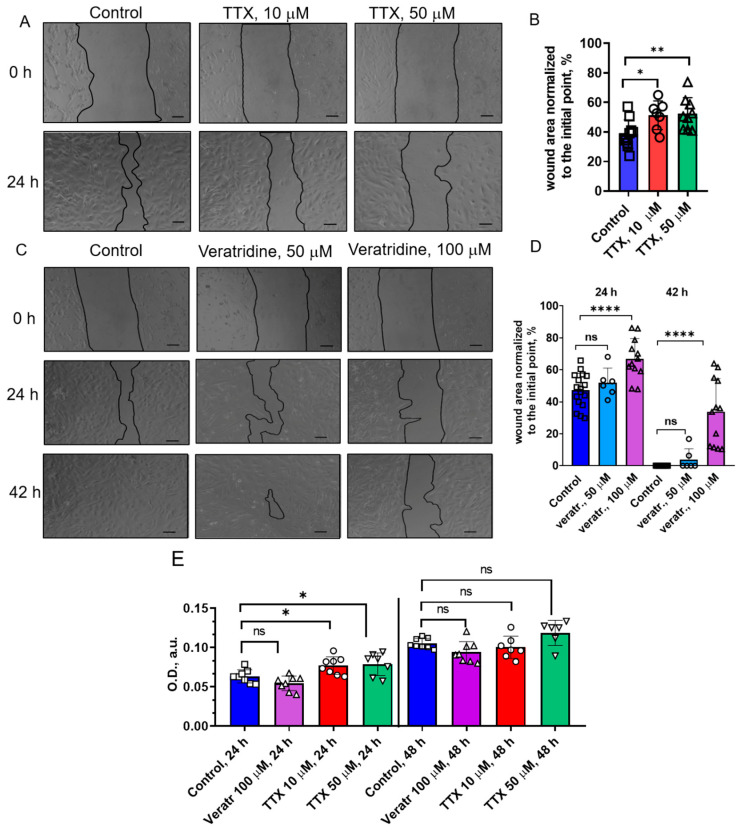
TTX and veratridine reduce the migration potential of eMSCs in 2D culture. Photographs of experimental wounds in the control and in the presence of different concentrations of TTX (**A**) or veratridine (**C**) in the culture medium at (0 h), 24 h, and 42 h after the start of the experiment. 10× objective, scale bar: 100 μm. (**B**,**D**) Quantitative analysis of the experimental wounds’ healing rate. Wound area values (in %) after 24 h, 42 h were normalized to the initial wound area of each experiment (0 h). Data were compared using an unpaired Student’s *t*-test (n = 6–16). (**E**) Viability and proliferation of eMSCs (MTT) in the control (0.2% DMSO) and in the presence of veratridine (100 μM) or TTX (10 and 50 μM) after 24 and 48 h. Data were compared to control values using the ANOVA statistical test. Data are presented as mean values ± S.D, * (*p* < 0.05), ** (*p* < 0.01), **** (*p* < 0.0001), ns—non significant.

The effect of TTX and veratridine on eMSCs in 2D culture was studied using the experimental “wound” healing method using live-cell imaging. Figure 3A shows the results of the wound healing experiment in the control and in the presence of the TTX at the beginning of the experiment (0 h) and after 24 h. Figure 3B shows the quantification of the results: the wound area values (in %) are normalized to the initial areas for each experimental condition (0 h). We found that TTX reduces the migration ability of eMSCs compared to the control experiment. In the presence of TTX at concentrations of 10 μM and 50 μM for 24 h, the wound area remained wider, with normalized values of 51.26 ± 3.67% and 52.28 ± 3.64% from the initial area, respectively, compared to the control (38 ± 3.06%). It should be noted that the TTX concentrations used here were significantly higher than in the electrophysiological experiments.

The effect of veratridine on the migratory potential of eMSCs was studied at concentrations from 5 to 100 μM. Interestingly, at low concentrations (5 μM), it did not affect the cell properties, but significant differences in eMSC migration were found in the presence of veratridine in concentrations up to 50 μM compared to the control (in the presence of the appropriate amount of DMSO). Figure 3A shows the results of the experiment with experimental wound healing under control conditions and in the presence of veratridine (50 μM and 100 μM) at the beginning of the experiment (0 h), and after 24 h and 42 h. Veratridine (100 μM) reduced the rate of wound healing (after 24 h—66.96 ± 3.65%, after 42 h—33.74 ± 6.00% from the initial area) compared to the control (after 24 h—47.21 ± 2.57%, after 42 h—0%, the wound had completely healed). No statistical difference was found between the area values in the control and in the presence of 50 μM veratridine (after 24 h—51.98 ± 3.75%; after 42 h—3.86 ± 2.79%).

Potentially, the decrease in cell migration could be due to the loss of cell viability or proliferation caused by veratridine or TTX treatments. To address this possibility, we tested the effect of veratridine and TTX on the metabolic activity of eMSCs using the MTT assay. According to the obtained results, veratridine does not affect the viability and proliferation of mesenchymal stem cells after 24 and 48 h, while TTX even slightly enhances them only after 24 h. Thus, it can be concluded that the decrease in the eMSCs migration properties was caused by the action of the studied reagents on cell motility and was not the result of a decrease in viability or proliferation.

#### 3.3.2. TTX and Veratridine Enhance the Migration Potential of eMSC Spheroids

**Figure 4:**
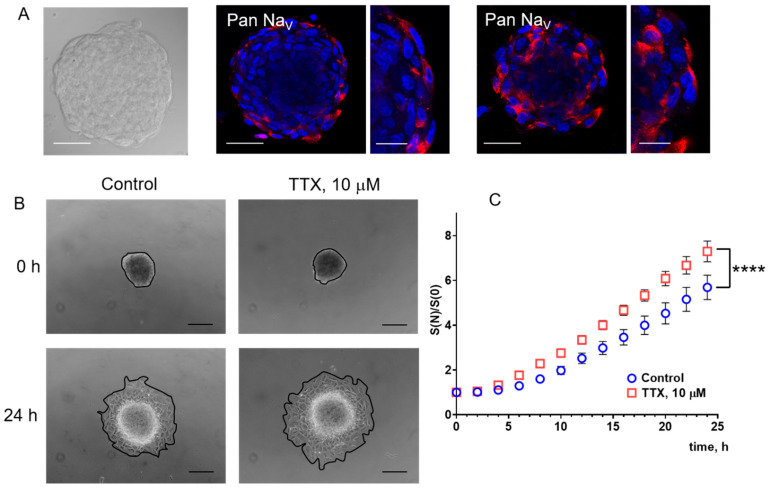
TTX increases the migration potential of eMSCs in 3D culture. (**A**) Immunofluorescence staining of NaV (Pan) proteins in the spheroids (red); cell nuclei were stained with DAPI (blue). Confocal images of the representative spheroid in the transmitted light and after the excitation at the wavelengths 543 (for Cy3 dye) and 405 (for DAPI), focusing near the center of the 3D structure and near the cover glass (from the bottom plane, spheroid attachment point). The scale bar is 50 μm. A 2.5× zoom is presented on the right of each image. The scale bar is 20 μm. (**B**) Photographs of spheroids formed from eMSCs in the transmitted light in the control and in the presence of 10 μM TTX in the culture medium at (0 h) and 24 h after the start of the experiment. Objective 10×, scale bar 200 μm. (**C**) Quantitative analysis of the spreading rate (SN/S0) of the spheroids. The areas of each spheroid (see black lines making the spheroid borders in (**B**)) are normalized to the initial areas at the beginning of the experiment (0 h). Data were compared using the Wilcoxon test and are presented as mean values ± S.E.M. **** (*p* < 0.0001), n = 11 in the control, n = 10 in TTX.

The expression of NaV in 3D eMSC culture (spheroids) was confirmed using immunofluorescence staining with Anti-Pan NaV antibodies, as in the 2D culture (Figure 4A). It should be noted that no staining of NaV or nuclei was observed in the center of the spheroid. This may be due to the inability of the dyes to penetrate the entire cell mass at the same rate as in a 2D culture, as we used the same immunofluorescence staining protocol.

**Figure 5:**
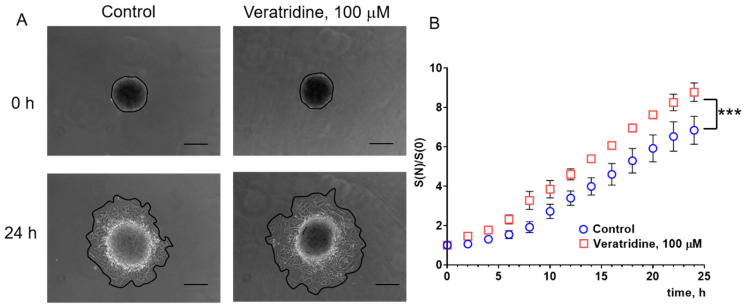
Veratridine increases the migration potential of eMSCs in 3D culture. (**A**) Photographs of spheroids formed from eMSCs in the transmitted light in the control and in the presence of 100 μM veratridine in the culture medium at (0 h) and 24 h after the start of the experiment. Objective 10×, scale bar 200 μm. (**B**) Quantitative analysis of the spreading rate (Sn/S0) of the spheroids. The areas of each spheroid (see black lines making the spheroid borders in (**A**)) are normalized to the initial areas at the beginning of the experiment (0 h). Data were compared using the Wilcoxon test and are presented as mean values ± S.E.M, *** (*p* < 0.001), n = 5 in each group.

In the next series of experiments, we studied the action of TTX and veratridine on the spreading rate of eMSCs from the spheroids (Figure 4 and Figure 5). Formed spheroids were seeded to the 24-well plate (one spheroid per well) in a fresh cell culture medium and allowed to attach to the plastic surface for 2 h. Photographs of the spheroids were taken every 2 h in the transmitted light for 24 h. The spread area of each spheroid at the experimental time point (S(N), where N is image acquisition time from the start of the experiment) was calculated separately and normalized to its initial area (S(0)). Surprisingly, in 3D culture, we observed the opposite effect of reagents on the migration properties of eMSCs compared to 2D culture. TTX (10 μM) significantly increased the spreading rate of the spheroids: from S(N)/S(0) 5.69 ± 0.55 in the control to 7.30 ± 0.46 in the presence of TTX after 24 h from the beginning of the experiments. Veratridine (100 μM) also increased the spreading rate of the spheroids from 6.84 ± 0.71 in the control to 8.77 ± 0.47 in the presence of veratridine.

## 4. Discussion

In this work, we studied the functional expression of voltage-gated sodium channels (NaV) in human endometrial MSCs and the role of these channels in cell motility. Using RT-PCR analysis, we identified the expression of almost all pore-forming α- and channel-modulating β-NaV subunits in eMSCs. Then, we detected channels activated by membrane depolarization of eMSCs in the whole-cell patch-clamp configuration. Inhibitory analysis revealed that the channels are TTX-sensitive and can be modulated or blocked by varying concentrations of veratridine. Using a wound healing assay, we found that TTX and veratridine reduce the migration properties of the cells in 2D culture but have the opposite effect on the spheroids, increasing cell spreading rate in 3D culture.

NaV channels, which are typical for nerve and muscle cells, were previously found in different non-excitable cells, including stem cells. Mainly, these works investigated the non-canonical functions of NaV, which are not associated with the conductive properties of channels. Most studies were devoted to identifying the role of NaV channels in various cancer cell lines such as breast, colon, prostate and ovarian cancer cells [9,23,24,25,26,27], where channel expression was positively or negatively correlated with cell proliferation, migration and increased risk of metastatic potential. Interestingly, both α- and β-subunits have been shown to be involved in the potentiation of cancer growth, metastasis, and angiogenesis [9,26,28]. At the same time, limited data are devoted to the study of NaV in stem cells. Firstly, the function of voltage-activated Na^+^ channels in hMSCs derived from the bone marrow was found. The authors showed that inward currents were TTX sensitive (to the concentrations 50–100 nM) and mRNA of hNE-Na (responsible for INaTTX), but not SCN5A, is present in the cells [29]. Enhanced expression of almost all α- and β-NaV subunits in human neural progenitor cells (hNPCs) was detected after differentiation, suggesting the important role of NaV during neuronal maturation in vitro [30]. In the other work on hMSCs from bone marrow, TTX at a concentration of 5 μM blocked inward currents elicited by depolarization and induced MSC proliferation but inhibited migration of the cells through transwells [31]. In this paper, the involvement of NaV in the migration properties of MSCs was first observed, and our results on 2D culture supported these data. We also registered the activity on NaV channels in eMSCs. However, in the literature, the contradictory result was shown, where despite the expression of SCN8A (NaV 1.6) detected in human induced pluripotent stem cells (iPSCs) by transcriptomic analysis and RT-PCR, NaV current activity was not found [32]. Such a difference in the results may be due to the various origins of the cells, the sources from which they were obtained and their functions.

We found functionally active voltage-gated Na^+^ channels in the plasma membrane of eMSCs; however, it is unclear why these channels might function in non-excitable cells if the main role of the channels is devoted to the generation and propagation of electrical signals. They can be activated if the membrane potential (Vm), the difference in the electrical charge between the cytosol and the extracellular environment, reaches certain negative (hyperpolarized) values. Vm is determined by the distribution and differential permeability of major ions, including K^+^ and Na^+^, and varies significantly (−10 to −90 mV) in various cell types, depending on their functions. Typically, the Vm of fast proliferating cells, including cancer cells or fibroblasts (MSCs are fibroblast-like cells), is depolarized (−10 to −30 mV) and insufficient to activate the channels. However, if cell proliferation slows down, which is characteristic for eMSCs in 3D culture [4], the concentration of the major ions changes, and Vm values can reach −60 to −70 mV [33]. Thus, channels may be expressed in the cells but not function until the concentration of Na^+^ ions reaches certain values.

**Figure 6:**
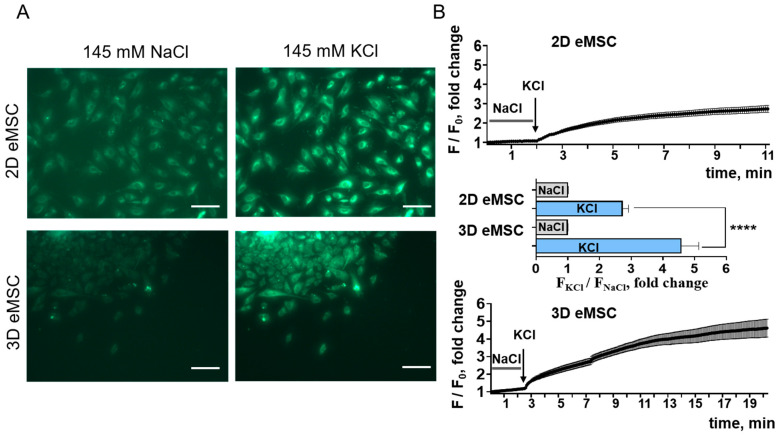
Quantification of the difference between resting and zero membrane potential in 2D culture and cells spreading from spheroids (3D) using DiBAC4(3) fluorescence. (**A**) Representative images of 2D and 3D eMSCs in Na^+^-based extracellular solution (“145 mM NaCl”) and after equivalent replacement of Na^+^ to K^+^ (“145 mM KCl”). The scale bar is 100 µm. (**B**) The dynamics of the changes in DiBAC4(3) fluorescence in 2D and spheroids. F_KCl_/F_NaCl_ of 3D eMSCs is significantly higher compared to 2D eMSCs, which indicates more hyperpolarized membrane potential. **** *p* < 0.0001. Also note the longer time needed for DiBAC4(3), which is a slow-response probe, to reach steady fluorescent state in 3D eMSCs, which is also an indication of more hyperpolarized potential.

To support this hypothesis, we assessed and compared the differences between resting membrane potential in 2D cell culture and 3D eMSC (cells spreading from spheroids) using potential-sensitive DiBAC4(3) dye (see Section 2.8). DiBAC4(3) cell fluorescence was measured in a standard physiological-like Na^+^-based solution, and then the cells were depolarized to 0 mV by the equal replacement of Na^+^ to K^+^ (Figure 6A,B). Although our measurements only show relative changes in fluorescence, not absolute Vm values, the quantification of F_KCl_/F_NaCl_ allowed the detection of significant differences between 2D and 3D cultures: the higher ratio indicates more hyperpolarized membrane potential in cell spheroids. The major limitation is that the membrane potential of the cells in the intact spheroids could not be properly measured using this technique. Therefore, to measure the membrane potential, we used the same time of eMSC spread from spheroids (24 h) as in the experiments studying the effect of our agents (TTX, veratridine) on the area of cell spread from spheroids.

Since we found the mRNA of almost all NaV subunits in eMSCs except NaV 1.3 and NaV β2, it is important to define possible α-subunits that form functionally active channels in these cells. We found that voltage-gated currents were blocked by the TTX concentrations lower than 30 nM; therefore, NaV 1.5, 1.8 and 1.9 can be excluded from the list of possible candidates as they are blocked by higher TTX values (IC50 NaV 1.5 = 5.7 µM, IC50 NaV 1.8 = 59 µM, IC50 NaV 1.9 = 40 µM) [22]. According to the literature, NaV 1.4 and NaV 1.5 can also be excluded, since their activation is observed at more negative potentials (−40–−60 mV) [22] than those recorded in our studies on eMSCs (−10–−20 mV). Thus, based on the electrophysiological properties, the following channel repertoire could be proposed: NaV 1.1, NaV 1.2, NaV 1.6 and NaV 1.7, and the activity of these channels could contribute to the observed effect of TTX on eMSC migration. However, the current pharmacological narrowing is insufficient for exact NaV channel identification. Also, the role of β-subunits should be taken into account. As β-subunits are not pore-forming and they only modulate channel activity, it is uncertain which β-subunits are involved in the regulation of NaV in eMSCs. According to the literature, both α- and β-NaV subunits may have non-canonical channel functions, including participation in the regulation of cell migration and proliferation (see, for example, the review by [5]). Therefore, it is possible that the found effects of TTX and veratridine on eMSC migration may be associated with the involvement of one or several α- and/or β-NaV subunits in the regulation of the cell reactions. In our study, the exact NaV isoforms responsible for the observed migratory effect remain unidentified and require further investigation in future studies.

In addition, we studied the effect of the alkaloid neurotoxin veratridine, which is used as a NaV channel agonist preventing channel inactivation and reducing peak current by shifting the potential toward hyperpolarization to activate the channel, on voltage-dependent current [34]. We found that veratridine in the lower concentrations (up to 40 μM) blocked voltage-gated currents, whereas 100 μM and above prevented channel inactivation. This effect, where veratridine acts as an antagonist, is unusual but has been previously reported in the literature. Experiments on Chinese hamster ovary (CHO) cells transfected with rat NaV 1.4 showed that veratridine can function as either an activator or inhibitor of NaV, and its action depends on the stimulation protocol [35]. A concentration-dependent dual action of veratridine has been shown on the functioning of NaV 1.6 expressed in freshly isolated murine vas deferens myocytes: veratridine at the concentrations from 1 to 10 µM increased peak current amplitude, while higher concentrations (≥30 µM) inhibited inward Na+ currents [36].

As we mentioned above, there are few data about the involvement of NaV in the migration properties of stem cells. We have shown that inhibition of NaV by TTX (10 and 50 µM) reduced the migration properties of 2D eMSC cultures. The same inhibitory TTX effect on the motility (at low µM concentrations) has been shown on MSCs derived from the bone marrow [31], on human prostate cancer cell lines [37] and on breast cancer cells, including the MDA-MB-231 cell line [23]. The authors suggest that the upregulation of NaV channel activity could potentiate the migratory and invasive processes of the cells due to different mechanisms, including protein–protein interplay with extracellular matrix or cytoskeletal components. It should be emphasized that both our work and other studies investigating NaV’s contribution to cell migration use TTX concentrations significantly higher than those used in electrophysiological experiments. Such high TTX values may carry a risk of unwanted side effects that could influence the obtained results.

We also found that veratridine (100 µM) inhibited the migration of eMSCs in 2D culture. Although veratridine is most often used as a NaV activator [34], in our experiments, it primarily inhibited channels. Consequently, its effect on migration was similar to that of TTX. There are almost no data about the effect of veratridine as a NaV modulator on the migration properties of non-excitable cells. Only one article mentioned that veratridine had no effect on the migration of prostate cancer cells [38]. Interestingly, in several relatively new studies, veratridine was used as an activator of the tumor-suppressor protein UBXN2A that is involved in the regulation of the mTORC2 signaling cascade. Veratridine decreased migration as well as metastatic functions of colon cancer cells [39]. Veratridine was also found to reduce the growth rate and migration of spheroids (3D culture) and suppress colon cancer cell migration and invasion, suggesting the use of veratridine as a promising anticancer drug [40], including its nanoparticle delivery to the colorectal cancer cells [39].

Our results demonstrated opposite effects of both agents (TTX and veratridine) on the migration properties of eMSCs cultured in 2D and 3D conditions. As we noted previously, human eMSCs assembled into 3D structures are more suitable for clinical studies than monolayers cultured in a 2D configuration. There is ample evidence that the transition from 2D to 3D culture results in significant changes in cell phenotype (including cell size and shape, cytoskeleton structure, expression level and functioning of ion channel genes) and behavior, which may explain the difference between our data on 2D and 3D eMSCs. Importantly, a recent study from our laboratory demonstrated the differences in ion channel regulation of cell migration between 2D and 3D eMSC cultures: selective inhibition of BK channel activity had no effect on 2D eMSCs, whereas the reactivation of 3D eMSC spheroids was significantly reduced [41]. Finding the reasons that lead to opposite effects of TTX and veratridine on 2D and 3D eMSCs may be the subject of our next study. Another study revealed specific changes in Na^+^ and K^+^ ion homeostasis in 2D and 3D cell cultures, where 2D eMSCs have a higher Na^+^ concentration and are more proliferative compared to 3D spheroids, which restore a low Na^+^ concentration and a high K^+^/Na^+^ ratio and do not proliferate [42]. The authors confirmed the role of the Na/K pump in changes in ion homeostasis, but it is possible that NaV or other Na-selective channels may also affect these changes.

It also cannot be ruled out that the effects of TTX and veratridine on the migration properties of eMSCs are not directly related to the functioning of the channels, but their action is determined by non-conductive channel properties. It may involve the interactions of the channels with various factors, including cellular mechanics (adhesion molecules or cytoskeletal proteins), the involvement of channels in the control of cell volume during migration, their interaction with other ion channels, or the activity of some signaling cascades like mTORC2. The exact regulatory mechanism remains to be elucidated.

## 5. Conclusions

Taken together, our data suggest that NaV channels are expressed in human eMSCs and play an important role in the regulation of stem cell migration; this regulatory mechanism significantly depends on the culture conditions of MSCs. The possibility of different or opposing effects of various agents in 2D and 3D cell cultivation should be taken into account before they are applied in tissue engineering or regenerative medicine.

## Figures and Tables

**Table 1:** List of primer sequences used for RT-PCR.

Gene	NaV Subunit	Forward Primer, 5′-3′	Reverse Primer, 5′-3′	Length, bp
*SCN1A*	NaV 1.1	TTCATGGCTTCCAATCCTTC	TAGCCCCACCTTTGATTTTG	178
*SCN2A*	NaV 1.2	GCCAGCTTATCAATCCCAAA	TCTTCTGCAATGCGTTGTTC	192
*SCN3A*	NaV 1.3	CAAAGGGAAGATCTGGTGGA	AAAGGCCAATGCACCACTAC	115
*SCN4A*	NaV 1.4	TCAACAACCCCTACCTGACC	ACGGACGAGTTCCCATCATA	148
*SCN5A*	NaV 1.5	CACGCGTTCACTTTCCTTC	CATCAGCCAGCTTCTTCACA	208
*SCN8A*	NaV 1.6	CGCCTTATGACCCAGGACTA	GTGCCTCTTCCTGTTGCTTC	247
*SCN9A*	NaV 1.7	GGCTCCTTGTTTTCTGCAAG	TGGCTTGGCTGATGTTACTG	196
*SCN10A*	NaV 1.8	ACCTGGTGGTGCTTAACCTG	TGCTGAAGAAGCTGCAAAGA	168
*SCN11A*	NaV 1.9	CTGTGGTCCTGGTCATTGTG	TGCATTCGCTTCTTGCATAC	233
*SCN1B*	β1	ACTGGTGTCCTCAGCCTG	AGGTCTCAGCGTTGGTCTC	125
*SCN2B*	β2	CCGACTAACATCTCAGTCTCTG	GCAGGTACTGTGACCTCCAT	134
*SCN3B*	β3	GCTTCTCTCGTGCTTATCTAC	CTCCACCTCCTCTCTCTTCA	136
*SCN4B*	β4	TCGTTGATAGACTGGAAGAAGT	CAGCAGGATGAGGATGAGGA	96

## Data Availability

The original contributions presented in this study are included in the article. Further inquiries can be directed to the corresponding author.
